# NMR Crystallographic Investigation Coupled with Molecular Dynamics Simulations Reveals the Nature of Disorder in Chlorpromazine Hydrochloride Solvatomorphs

**DOI:** 10.1021/acs.molpharmaceut.5c00269

**Published:** 2025-07-19

**Authors:** Scott A. Southern, Vijith Kumar, Victor Terskikh, David L. Bryce, Andreas Brinkmann

**Affiliations:** † 6356 Metrology, National Research Council Canada, 1200 Montreal Road, M40, Ottawa, Ontario K1A 0R6, Canada; ‡ Department of Chemistry and Biomolecular Sciences, 6363 University of Ottawa, 10 Marie Curie Private, Ottawa, Ontario K1N 6N5, Canada

**Keywords:** solid-state NMR, NMR crystallography, disorder, pharmaceutical API

## Abstract

Chlorpromazine, a widely used antipsychotic medication formulated as a hydrochloride salt, has been a significant active pharmaceutical ingredient (API) for the treatment of schizophrenia for much of the last century. This work presents a comprehensive investigation into the nature of the structural disorder of chlorpromazine hydrochloride solvatomorphs using a combination of nuclear magnetic resonance (NMR) crystallography and molecular dynamics simulations. We focus on understanding the structural characteristics and stability of chlorpromazine hydrochloride and its hydrate, particularly the disorder in the dimethylaminopropyl side chain. This work provides a detailed analysis of the structural characteristics influencing this disorder, advancing the understanding of drug development and the design process for APIs that exhibit similar types of disorder.

## Graphical abstract

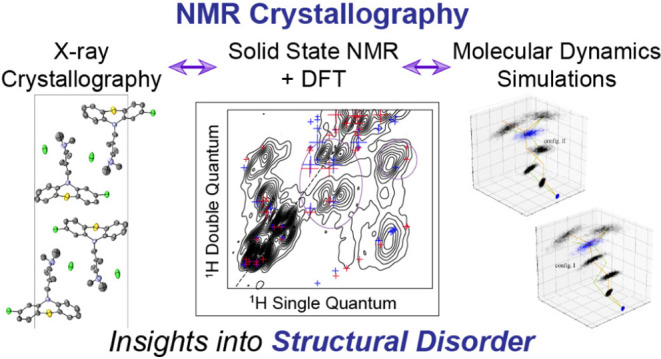

## Introduction

Chlorpromazine, an antipsychotic medication, is an API formulated as a hydrochloride salt and has long been used in both humans and animals.^1,2^ In 2020, chlorpromazine was even investigated as a possible antiviral treatment against COVID-19;^3–5^ although, recent clinical data have suggested that the drug is not associated with a decrease in COVID-19 related mortality.^6^ Even so, chlorpromazine has remained an important API for over 70 years in the treatment of psychotic ailments such as schizophrenia and bipolar disorder.^7^

The first known crystals of neutral chlorpromazine were obtained from ether^8^ and ethyl alcohol^9^ following conversion from the hydrochloride, with the crystallographic unit cell data published a few years later.^10^ In 1969, McDowell solved the crystal structure of chlorpromazine, confirming the *Pbca* space group.^11^ The crystal structure of chlorpromazine hydrochloride, shown in Scheme 1, was later solved by Dorignac-Calas and Marseau in 1972,^12^ confirming the *P*2_1_/*c* space group previously reported in 1965.^13^ Later, a hydrate of chlorpromazine hydrochloride was identified.^14^ Similar to the hydrochloride, this solvatomorph crystallizes in a *P*2_1_/*c* space group, but with two unique units of chlorpromazine HCl and one-half water molecule per each unit cell, resulting in a hemihydrate.

**Scheme 1:**
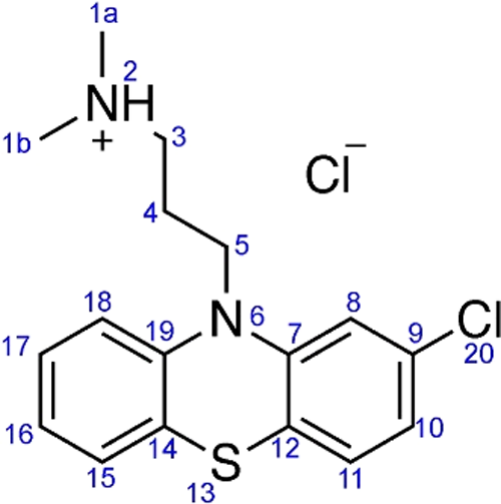
General Numbering Scheme for Chlorpromazine HCl^a^

a Hydrogen atoms are numbered according to the heavier atom to which they are bonded.

Crystallographic disorder exists in two main forms. Static disorder arises when a molecule adopts more than one molecular conformation due inter- or intramolecular forces to cause a molecule to adopt a range of molecular conformations. By contrast, dynamic disorder arises from fluctuations of structural properties such as motions of atoms or atomic groups in molecules . Unlike neutral chlorpromazine, the hydrochloride exhibits a high degree of disorder in the dimethylaminopropyl side chain, leading to uncertainty in its geometric structure.^12^

Precise knowledge of a solid API’s crystal structure is vital for predicting and understanding its physicochemical properties, chemical stability^15^ and bioavailability,^16^ and it is highly relevant for securing intellectual property rights. A key objective in pharmaceutical development is to identify crystalline forms that balance sufficient thermodynamic stability with desirable pharmaceutical properties, such as dissolution rate, shelf life and bioavailability, to mitigate the risk of “disappearing polymorphs,” which can compromise efficacy and marketability.^17^ Both static and dynamic disorder can significantly impact on pharmaceutical development.

Solid-state analytical methods are essential for understanding the chemical properties of solid materials. In combination with other analytical techniques such as powder X-ray diffraction (PXRD), single-crystal X-ray diffraction (SC-XRD), and *ab initio* calculations, solid-state NMR techniques can offer unique insights into subtle structural differences among polymorphs and solvatomorphs. While X-ray crystallography remains an essential tool for the analyzing pharmaceutical compounds, it can be challenging to achieve a complete and unambiguous structural analysis on compounds using this method alone. The field of NMR crystallography combines solid-state NMR, diffraction data, and computational chemistry to determine new crystal structures, validate or refine known crystal structures, and select structures among multiple polymorphs and solvatomorphs.^18–21^

While X-ray diffraction has been used to solve the crystal structure of chlorpromazine, questions remain regarding the exact structural arrangement in each form, particularly concerning the disorder in the 3-(dimethylamino)­propyl side chain. An earlier analysis of the molecular motion in chlorpromazine and chlorpromazine hydrochloride was carried out using NMR relaxation measurements^22^ establishing activation energies for the rotation of the methyl groups, and the translational motion of the 3-(dimethylamino)­propyl side chain. The investigation provided valuable insights into the methyl group motion but suggested that the side chain is relatively static, resulting only in an overall phase change at elevated temperatures.

Given that many pharmaceuticals are derived as hydrochloride salts, solid-state NMR studies of pharmaceuticals often focus primarily on ^35^Cl NMR^23–25^ due to the sensitivity of its chemical shielding and electric field gradient (EFG) tensor parameters to fluctuations in the local chemical environment.^26^ Recently, an NMR study demonstrated how dynamics and disorder in three pharmaceutical hydrochlorides could be inferred from ^13^C and ^35^Cl NMR spectra.^27^ Herein, we undertake a detailed multinuclear NMR study of chlorpromazine hydrochloride and its solvatomorph by combining solid-state NMR, density functional theory (DFT), and molecular dynamics simulations to gain deeper insights into the dynamics and stability of the chlorpromazine aliphatic side chain. This study serves as an example for analyzing APIs exhibiting comparable disorder, enabling a better understanding of which structural characteristics can affect disorder and how that disorder might affect the overall stability of an API. Current crystal structure prediction methods do not fully account for static or dynamic disorder, but additional knowledge of the structural features that influence disorder will advance drug development capabilities by introducing stronger design constraints.

## Materials and Methods

### Recrystallizations

A powdered sample of chlorpromazine hydrochloride (CPZ–HCl) was obtained from Sigma-Aldrich and used without further purification. To prepare crystals for single-crystal X-ray diffraction (SC-XRD), CPZ–HCl was dissolved in acetone and recrystallized by vapor diffusion of cyclohexane into the solution. The process was carried out in a sealed container under a constant nitrogen flow for several weeks yielding long needle-shaped crystals suitable for analysis.

A hemihydrate of chlorpromazine hydrochloride (CPZ–HCl·1/2H_2_O) was obtained by dissolving CPZ–HCl in a 1:10 solution of water and ethanol. The solution was left exposed to laboratory air for several weeks after which slab-like crystals formed. The sample was sealed in its mother liquor and stored at −25 °C to prevent degradation.

### Powder X-ray Diffraction

Powder X-ray diffraction data were collected using a Rigaku Ultima IV powder diffractometer operating at room temperature using Cu Kα_1_ radiation (λ = 1.54056 Å). A continuous scanner was used to carry out the measurements over a range of 2θ values of 5–60° in increments of 0.02°. The experimental PXRD patterns were compared to patterns calculated in Mercury, showing excellent agreement (Figure 1).

**Figure 1:**
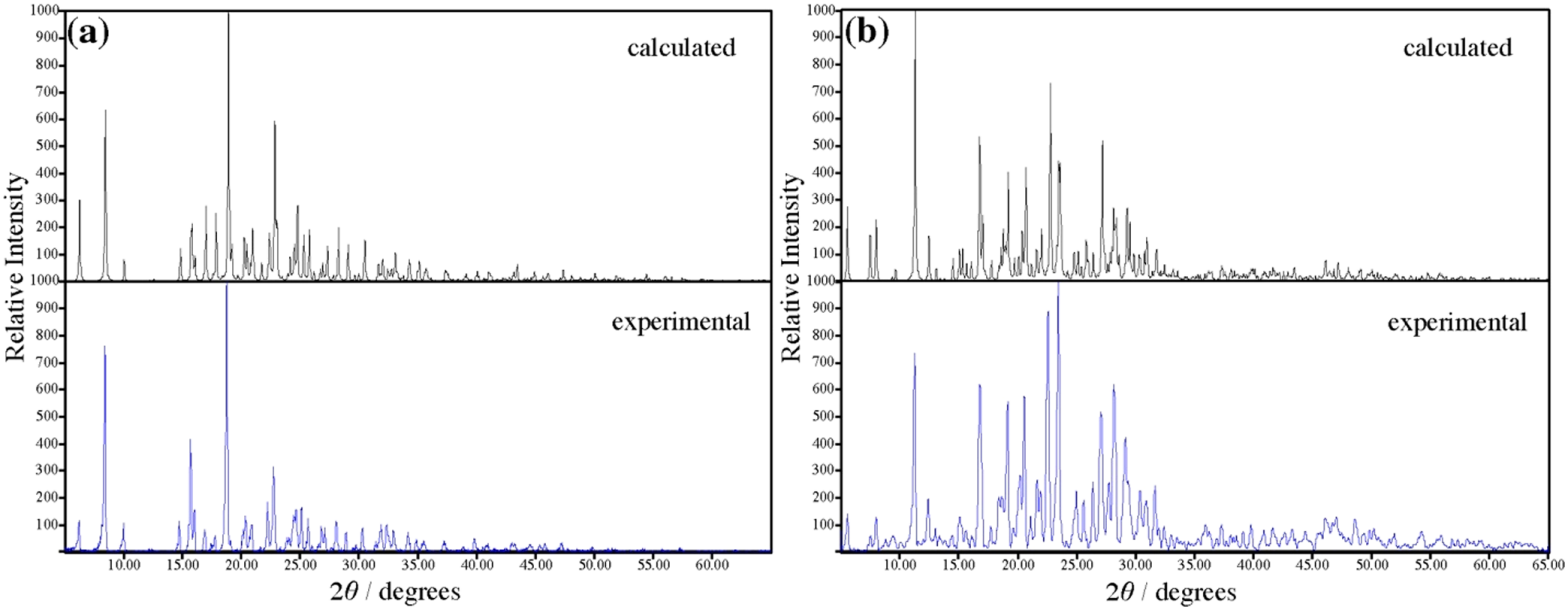
Experimental and calculated PXRD of (a) chlorpromazine hydrochloride and (b) chlorpromazine hydrochloride hemihydrate.

### Single Crystal X-ray Diffraction

A single crystal was mounted on a MiTeGen MicroMounts precision tool prior to data collection. X-ray data were collected on a Bruker Kappa Apex diffractometer equipped with Mo Kα radiation (λ = 0.7103 Å) and an APEX II CCD detector at 200 ± 2 K. The Bruker APEX III software package was used to collect and process the raw data. Crystal structures of chlorpromazine hydrochloride and chlorpromazine hydrochloride hemihydrate were solved using Olex2 ^28^ using the direct method and refined against F2 using SHELXL97.^29,30^ Non-hydrogen atoms were refined anisotropically, while hydrogen atoms were positioned geometrically. The alkyl side chain in CPZ–HCl shows larger thermal ellipsoids and positional disorder. PART instructions separated the disordered components, and occupancies were refined using the free variable and displacement parameter restraints (SADI and SIMU).

### Solid-State NMR Spectroscopy

The NMR tensor conventions used in this work are outlined as follows. The solid-state NMR spectra of isolated spin-1/2 nuclei primarily reflect the magnetic shielding interaction tensor. For half-integer quadrupolar nuclei, both the magnetic shielding and the quadrupolar interaction contribute significantly. The magnetic shielding tensor, ***σ***, is described by three principal components, *σ*_11_ , *σ*_22_ , and *σ*_33_ , ordered according to the Maryland Convention such that σ_33_ ≥ σ_22_ ≥ σ_11_ .^31^ This work adopts the Herzfeld–Berger convention^32^ to describe the magnetic shielding tensor. The isotropic magnetic shielding constant, σ_iso_ (in ppm), is calculated as

1 $$\sigma_{\mathrm{iso}}=\frac{\sigma_{11}+\sigma_{22}+\sigma_{33}}{3}$$

The anisotropy of the tensor is described by the span, Ω (in ppm), given by

2 $$\Omega =\sigma_{33}-\sigma_{11}$$

and the unitless skew, κ , given by

3 $$\kappa =\frac{\sigma_{11}+\sigma_{33}-2\sigma_{22}}{\Omega }$$

The isotropic chemical shift, δ_iso_ (in ppm), relates to the magnetic shielding by

4 $$\delta_{\mathrm{iso}}=\frac{\sigma_{\mathrm{ref}}-\sigma_{\mathrm{iso}}}{1-\sigma_{\mathrm{ref}}}$$

where σ_ref_ is the isotropic shielding of a suitable reference compound.

For quadrupolar nuclei (*I* > 1/2), the electrical quadrupole moment, *Q* (in mb), describes the asymmetric charge distribution within the nucleus, and couples with the electric field gradient (EFG) at the nucleus, represented by the principal components *V*_11_ , *V*_22_, and *V*_33_ , ordered |*V*_33_| ≥ |*V*_22_| ≥ |*V*_11_| .^33^ The quadrupolar coupling constant, *C*_Q_ (in MHz), is given by

5 $$C_{Q}=\frac{eQV_{33}}{h}$$

where *e* is the fundamental charge and *h* is Planck’s constant. The unitless asymmetry parameter (η_Q_) describes the deviation of the EFG tensor from axial symmetry and is given by

6 $$\eta_{Q}=\frac{V_{11}-V_{22}}{V_{33}}$$

Together, these parameters provide important insights into the local chemical environment around the quadrupolar nucleus.

All solid-state NMR experiments were conducted in external magnetic field strengths (*B*_0_) of 9.4 T (Larmor frequency, *ν*_L_ (^1^H) = 400.13 MHz) or 21.1 T (*ν*_L_(^1^H) = 900.13 MHz) on Bruker Avance III and Avance instruments, respectively. Unless otherwise noted, at 9.4 T, a wide-bore 3.2 mm triple resonance MAS probe was used at ambient temperatures, and at 21.1 T, a standard-bore 3.2 mm double resonance MAS probe was used. Samples were center-packed in zirconia rotors with Kel-F spacers and spun at the magic angle (54.74°), calibrated by maximizing rotational echoes in the ^79^Br free-induction decay (FID) of KBr.

^1^H–^13^C cross-polarization magic-angle-spinning (CP/MAS) experiments^34^ used ramped pulses^35^ and XiX^36^ or SPINAL64 ^37^ proton decoupling. Samples were spun at 12.5 kHz or 15–20 kHz at 9.4 and 21.1 T respectively, to avoid overlapping signals with spinning sidebands. All ^13^C MAS NMR spectra were referenced to adamantane (37.77 ppm relative to 1% TMS in CDCl_3_).^38^ 2D ^1^H–^13^C HETCOR experiments were acquired at 12.5 kHz MAS using e-DUMBO-12.5 ^39^ incorporating a ^1^H 90° pulse of 2.15 μs and a DUMBO pulse optimized to 24.4 μs, with a resolution of 0.2 μs. A recycle delay of 2 s was used while 200 saturation pulses were applied on the ^1^H channel.

^35^Cl MAS spin–echo experiments ((π/2)−τ–(π)−τ) were acquired at 21.1 T (*ν*_L_(^35^Cl) = 88.19 MHz), using a 3.2 mm Bruker triple resonance probe operating at MAS rates of 20–22 kHz. ^35^Cl chemical shifts were referenced to solid NaCl (−41.11 ppm). Central transition (CT) selective ^35^Cl pulses with duration 3 μs were applied, and the echo delay was set to 45.5 and 41 μs for 20 and 22 kHz spinning speeds, respectively. Experiments were allowed to run for 16–29 h, depending on the signal intensity, using 1 s recycle delays.

All static ^35^Cl NMR experiments were acquired at 21.1 T using a 7 mm home-built two-coil design H/X probe using radiofrequency (RF) coils covering tuning ranges of 69–87 MHz and 85–101 MHz. Using the same probe, static ^14^N NMR experiments were acquired at 21.1 T using an RF coil capable of a 61–70 MHz tuning range.

Powdered samples were center packed between layers of Teflon tape in glass vials with an outer diameter of ∼7 mm. ^35^Cl WURST-QCPMG^40,41^ experiments were collected using the variable-offset cumulative spectral acquisition (VOCS) method^42^ with frequency steps of 5 kHz. 50 μs pulses were swept over 2 MHz with spikelets separated by 20 kHz. 8,192 scans were collected during each acquisition step using recycle delays of 0.2–0.5 s. We note that in the case of chlorpromazine hydrochloride, we could not acquire any signal due to the unusually short transverse relaxation time (*T*_2_) for this sample. We instead acquired a partial VOCS static quadrupolar echo spectrum for these compounds to observe the most important spectral features allowing NMR parameter extraction. These experiments were acquired using two 90° pulses separated by an echo delay of 25 μs, followed by a refocusing time of the same length. Each scan was acquired following a 0.1 or 0.2 s delay for 65,000 scans per experiment. Each spectrum was acquired with transmitter offsets stepped by 0.1 MHz, which were then combined to form a skyline projection of the NMR signal around each singularity. The middle of the powder pattern was omitted because acquiring the entire powder pattern was impractical and ultimately unnecessary. ^14^N WURST-QCPMG spectra were obtained using the VOCS method and frequency steps of 200 kHz, 50 μs pulses were swept over 2 MHz with spikelets separated by 5 kHz and 8,192 scans were collected during each acquisition step using recycle delays of 0.2s.

Static ^35^Cl quadrupolar echo experiments ((π/2)−τ–(π/2)−τ) were acquired using 5 μs CT-selective ^35^Cl pulses, and 64 μs echo delays. Experiments were allowed to run for 6 to 29 h, depending on the signal intensity, using 2 s recycle delays.

### Quantum Chemical Calculations

Plane-wave DFT calculations were carried out in CASTEP using Materials Studio version 19.1.^43^ The revised Perdew–Burke–Ernzerhof (RPBE)^44^ functional was used with on-the-fly generated (OTFG) scalar ZORA ultrasoft pseudopotentials. The plane-wave cutoff energy was converged to 600 eV and a minimum *k*-point^45^ spacing of 0.07 Å^–1^ was used for geometry optimizations and NMR property calculations. Geometry optimizations were carried out using the minimization approach of Broyden, Fletcher, Goldfarb, and Shanno (BFGS) with unit cell dimensions held at their experimental values.^46^ Two-body dispersion corrections (Grimme D2) with a reparameterized damping function (*s*_6_ = 1.0; *d* = 5.0) were used.^47^ The gauge-including projector augmented wave (GIPAW) approach was used to compute magnetic shielding tensors and NMR parameters were extracted using EFGShield version 4.5 ^48^ and MagresView.^49^ While more recent schemes such as PBE + D4 or PBE + MBD may offer improved accuracy, RPBE + D2 has demonstrated consistent reliability for organic pharmaceutical crystals in prior NMR-crystallography benchmarks, discussed *vide infra*.

Molecular dynamics (MD) calculations were performed in three steps: (1) fully optimize the geometry I and II of CPZ–HCl (see below), (2) perform MD runs on each molecule until thermal equilibrium is reached, and finally, (3) perform the actual MD calculations on each molecule. Calculations were carried out in CASTEP using Materials Studio version 19.1. For all three steps, the generalized gradient approximation with the PBE functional and ultrasoft pseudopotentials with a cutoff energy of 240 eV and a minimum *k*-point spacing of 0.08 Å^–1^ were chosen. The integration time-step was set to 0.5 fs, and the canonical (NVT) ensemble held at a constant temperature of 315 K employing a Langevin thermostat was used. NVT conditions were chosen to preserve the experimental unit-cell dimensions, enabling direct comparison of interatomic distances with NMR-derived parameters. The MD run to thermal equilibrium lasted a total of 0.35 ps using a thermostat time constant of 0.05 ps. The total simulation time of the actual MD calculations was 5.0 ps, where a thermostat time constant of 0.1 ps was used, resulting in a mean temperature of 313.4 K with a standard deviation of 18.2 K.

The transition state (TS) search was carried out using in CASTEP using Materials Studio version 19.1. The calculation was separated into three stages featuring reaction configurations (reactant: A­(*P*2_1_/*c* → *P*1); TS: (P1); and product: B­(*P*2_1_/*c* → *P*1)). The same protocol as used for the geometry optimization was implemented here. In short, the revised RPBE functional was used with OTFG scalar ZORA ultrasoft pseudopotentials. The plane-wave cutoff energy was 700 eV and a minimum *k*-point spacing of 0.07 Å^–1^ was used for geometry optimizations. Two-body dispersion corrections (Grimme D2) with a reparameterized damping function (*s*_6_ = 1.0; *d* = 5.0) were used. One unit cell containing four molecules was used with its dimensions held at their experimental values.

## Results and Discussion

### X-ray Crystallography

Dorignac-Calas and Marseau first reported the complete crystal structure of metastable CPZ–HCl in 1972,^12^ describing a monoclinic *P*2_1_/*c* space group with one molecule of chlorpromazine and one chloride ion in the asymmetric unit. The authors noted thermal disorder at positions C4 and C5 in the 3-(dimethylamino)­propyl side chain, leading to a distorted structure. ^1^H solution NMR data suggested that the position of the side chain can adopt three structural conformations, perpendicular or planar to the phenothiazine ring, or folded back onto itself.^50^ Solid-state NMR relaxation studies proposed that the motional freedom of the side chain leads to distinct phase transitions at elevated temperatures.^22^ Furthermore, upon wet granulation, the tableting characteristics were significantly improved, attributed to changes in the crystal lattice enabling interparticulate bonding.^51^ To better understand the molecular factors contributing to these significant macroscopic property changes, we sought to determine whether the side chain disorder (static or dynamic), plays a role.

We began by recrystallizing CPZ–HCl and CPZ–HCl·1/2H_2_O to better characterize the 3-(dimethylamino)­propyl side chain. Figure 2a shows the ORTEP diagram of CPZ–HCl, while the crystal packing is shown in Figure 2b. Crystallographic data and refinement parameters are in Table 1. In agreement with prior observations,^12^ we found a *P*2_1_/*c* space group. The side chain disorder manifests by two unique configurations, I and II (Figure 2a). Assuming static disorder with an equally distributed population of configurations, we measured significant differences in dihedral angles. Between I and II, the dihedral angles are N6–C5–C4^I^–C3^I^ = 177.8(9)° and N6–C5–C4^II^-C3^II^ = 86.7(6)°. These results improve the previously reported structure of the distorted side chain.^12^

**Figure 2:**
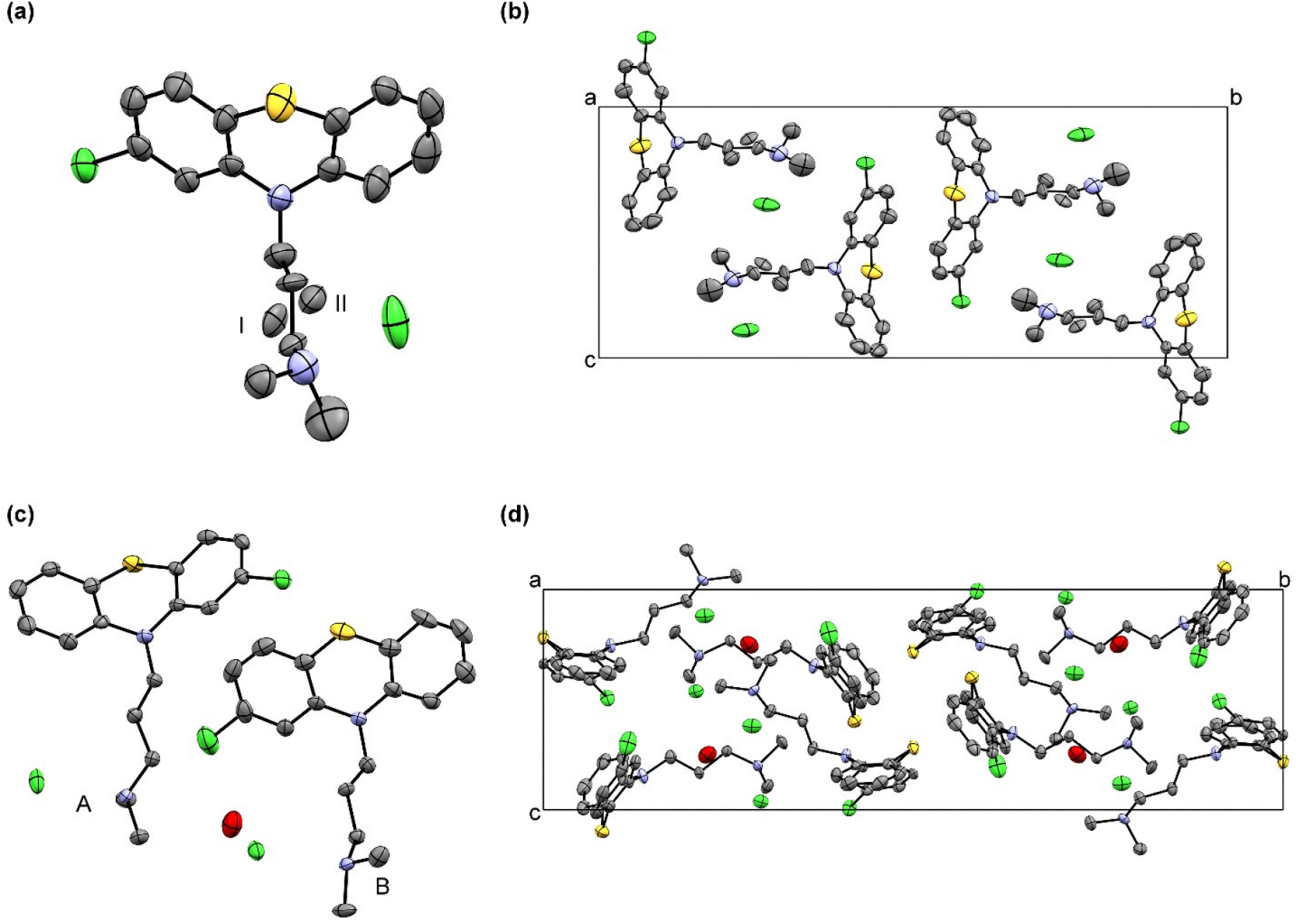
Molecular representation with thermal ellipsoids of (a) CPZ–HCl and (c) CPZ–HCl·1/2H_2_O, and their packing diagrams in (b) and (d), respectively. In (a), the disorder in the 3-(dimethylamino)­propyl side chain is shown with two unique configurations (I and II). Hydrogen atoms are omitted for clarity.

**Table 1:** Crystallographic Data and Structure Refinement Parameters

Compound	CPZ–HCl	CPZ–HCl·1/2H_2_O
Empirical formula	C_17_H_19_Cl_2_N_2_S	C_34_H_42_Cl_4_N_4_OS_2_
Formula weight	354.60	728.63
Temperature (K)	200 (2)	200 (2)
Crystal system	Monoclinic	Monoclinic
Space group	*P*2_1_/*c*	*P*2_1_/*c*
*a* (Å)	5.6386(8)	11.9091(5)
*b* (Å)	28.154(4)	31.7008(13)
*c* (Å)	11.3515(16)	9.6702(4)
α (°)	90	90
β (°)	97.934(4)	99.073(2)
γ (°)	90	90
Volume (Å^3^)	1784.8(4)	3605.1(3)
*Z*	4	4
ρ_calc_ (g/cm^3^)	1.320	1.342
μ (mm^1^)	0.478	0.478
*F*(000)	741.0	1528.0
Crystal size (mm^3^)	0.28 × 0.26 × 0.05	0.16 × 0.09 × 0.05
Radiation	Mo Kα (λ = 0.71073)	Mo Kα (λ = 0.71073)
2θ range (°)	3.9 to 55.082	2.57 to 51.994
Reflections collected	17,493	22,221
Independent reflections	4122	7048
*R*_int_, *R*_sigma_	0.0806, 0.0852	0.0962, 0.1272
Data/restraints/parameters	4122/75/224	7048/0/413
Goodness-of-fit on *F* ^2^	0.991	0.972
Final *R* indexes [*I* ≥ 2σ(*I*)]	*R*_1_ = 0.0598	*R*_1_ = 0.0667
	w*R*_2_ = 0.1503	w*R*_2_ = 0.1448
Final *R* indexes [all data]	*R*_1_ = 0.1482	*R*_1_ = 0.1533
	w*R*_2_ = 0.1889	w*R*_2_ = 0.1697
Largest diff. peak/hole (e Å^–3^)	0.73/–0.64	1.46/–0.53

We also solved the crystal structure of CPZ–HCl·1/2H_2_O (Figure 2c, d). Consistent with Klein and Conrad^14^ the space group is *P*2_1_/*c* and the unit cell dimensions closely matched earlier data. The critical difference is that the hemihydrate does not display 3-dimethylaminopropyl side chain disorder, yet, in both crystal structures, the tertiary amine forms a hydrogen bond with a nearby chloride anion.

We then carried out an NMR crystallographic study to understand the difference in side chain disorder between the two solvatomorphs. Plane-wave DFT geometry optimizations were carried out on modified CPZ–HCl structures containing configuration I or II. Quantum chemical calculations of NMR parameters can be significantly affected by differences in structural refinements resulting from the reparameterizations of the two-body dispersion correction schemes.^47,52^ In Grimme’s D2 model, the two-body dispersion energy depends on a Fermi-type damping function, where the steepness factor, *d*, determines the gradient of the universal damping function essential for accounting for short-range effects in the dispersion correction.^53^ An additional scaling parameter, *s*_6_, may be modified depending on the density functional used.^53–55^ Modifying *s*_6_ and *d* affects the structural geometries while maintaining a fixed unit cell.

Table 2 compares side chain geometries predicted by carrying out plane-wave DFT calculations by setting *d* to either 3.25 ^52^ or 5,^56^ and *s*_6_ to 1.0. When only one side chain configuration is used (I or II), each one independently relaxes to a distinct local potential energy minimum. Overall, the optimized structures agree well with experiment, with adjusting the steepness factor to *d* = 5, yielding slightly better agreement with the X-ray data. A notable exception is the N2–C3 bond length determined for configuration I, which is underestimated by DFT. Some structural differences also exist between our X-ray crystal structure and the geometries observed in the 1972 ^12^ crystal structure. For example, there are large differences in the torsional angles due to the resolution of disorder in our crystal structure, and the N2–C3 bonds differ by almost 0.1 Å. However, the N2–Cl bond length remains consistent at close to 3 Å for all structures, suggesting that this N–H–Cl contact may be an important anchor point for the side-chain.

**Table 2:** Comparison of Geometrical Features between the Previously Published Crystal Structure of Chlorpromazine HCl, Our Crystal Structure of the Same Compound, and a GIPAW DFT Geometry-Optimized Crystal Structure Using Grimme’s Dispersion Correction with Parameters *d* = 5 or 3.25 and *s*_6_ = 1.00

		Experimental			Calculated			
Atoms	Geometry	Dorignac^12^	This work (I)	This work (II)	*d* = 5; *s*_6_ = 1.00(I)	*d* = 5; *s*_6_ = 1.00(II)	*d* = 3.25; *s*_6_ = 1.00(I)	*d* = 3.25; *s*_6_ = 1.00(II)
N6–C5–C4–C3	Torsion [deg]	151.86	177.8(9)	86.7(6)	173.3	87.4	172.5	86.5
N2–C3–C4–C5	Torsion [deg]	126.74	179.5(8)	179.2(2)	175.1	177.6	175.8	177.7
N2–Cl	Length [Å]	2.931	2.962(4)		3.02	2.99	3.03	3.00
C9–Cl(A)	Length [Å]	1.74	1.739(4)		1.74	1.74	1.73	1.73
N6–C5 (A)	Length [Å]	1.484	1.465(6)		1.45	1.46	1.44	1.44
N2–C1a (A)	Length [Å]	1.514	1.475(7)		1.48	1.48	1.46	1.46
N2–C1b (A)	Length [Å]	1.512	1.470(7)		1.48	1.48	1.47	1.47
N2–C3	Length [Å]	1.588	1.582(9)	1.460(11)	1.49	1.49	1.48	1.48

The same calculations were formed for the crystal structure of CPZ–HCl·1/2H_2_O (Table 3), showing that setting *d* = 5 results in better agreement of structural angles and heavy-atom distances with experiment. For example, the N···O distances and angles, which reflect the position of the water molecules relative to the chlorpromazine side-chain region, are substantially preserved but with slightly better agreement when *d* = 5. On the other hand, the N···Cl^–^ distances do not change whether *d* is set to 5 or 3.25. Whether this agreement is retained when calculating the NMR parameters of ^13^C, ^35^Cl and ^15^N is discussed further in detail.

**Table 3:** Comparison of Geometrical Properties for Chlorpromazine HCl Hemihydrate from Previously Published Crystal Structure and GIPAW DFT Geometry-Optimized Crystal Structures Using Varied Reparametrizations of Grimme’s Two-Body Dispersion Correction

Atoms	Geometry	DUKTOS^14^	This work	*d* = 5; *s*_6_ = 1.00	*d* = 3.25; *s*_6_ = 1.00
N6–C5–C4–C3 (A)	Torsion [deg]	171.48	170.46	170.60	170.70
N6–C5–C4–C3 (B)	Torsion [deg]	179.55	179.29	179.88	179.65
N2–C3–C4–C5 (A)	Torsion [deg]	164.90	162.65	161.06	162.22
N2–C3–C4–C5 (B)	Torsion [deg]	166.60	166.88	168.41	171.93
N2(A)–O	Length [Å]	4.09	3.623	3.625	3.654
N2(B)–O	Length [Å]	4.19	4.219	4.217	4.238
N2(A)–Cl	Length [Å]	2.990	2.995	3.00	3.00
N2(B)–Cl	Length[Å]	3.027	3.052	3.06	3.05
N2(A)–O–N2(B)^1^	Angle [°]	132.56	132.72	132.65	131.68
N2(A)–O–N2(B)^2^	Angle [°]	85.04	86.77	86.35	87.89
C9–Cl(A)	Length [Å]	1.76	1.74	1.741	1.730
C9–Cl(B)	Length [Å]	1.73	1.739	1.744	1.731
N6–C5 (A)	Length [Å]	1.45	1.47	1.454	1.438
N6–C5 (B)	Length [Å]	1.44	1.459	1.452	1.437
N2–C1a (A)	Length [Å]	1.47	1.489	1.482	1.464
N2–C1b (A)	Length [Å]	1.47	1.481	1.484	1.467
N2–C1a (A)	Length [Å]	1.50	1.485	1.486	1.467
N2–C1b (A)	Length [Å]	1.49	1.484	1.484	1.468
N2–C3 (A)	Length [Å]	1.50	1.495	1.494	1.476
N2–C3 (B)	Length [Å]	1.51	1.493	1.494	1.477

### Solid-State NMR Spectroscopy

To understand how intermolecular interactions may influence the structural characteristics of the side chain , we applied NMR crystallographic principles beginning with ^13^C CP/MAS experiments acquired at 9.4 and 21.1 T. Figure 3a shows the stacked ^13^C CP/MAS spectra of chlorpromazine HCl, which exhibit narrow resonances consistent with a highly crystalline sample. Four peaks are observed between 20 and 60 ppm, corresponding to the side chain, although the CP/MAS data alone cannot confirm multiple side chain configurations. In contrast, the hemihydrate (Figure 3b) features more complex spectra due to the presence of two molecules of chlorpromazine, and thus more crystallographically distinct ^13^C sites. We note the broadening of the peak at ∼132 ppm in both compounds arising from indirect quadrupolar effects from the covalently bound chlorine, which is partially averaged at 21.1 T, especially in the hemihydrate.

**Figure 3:**
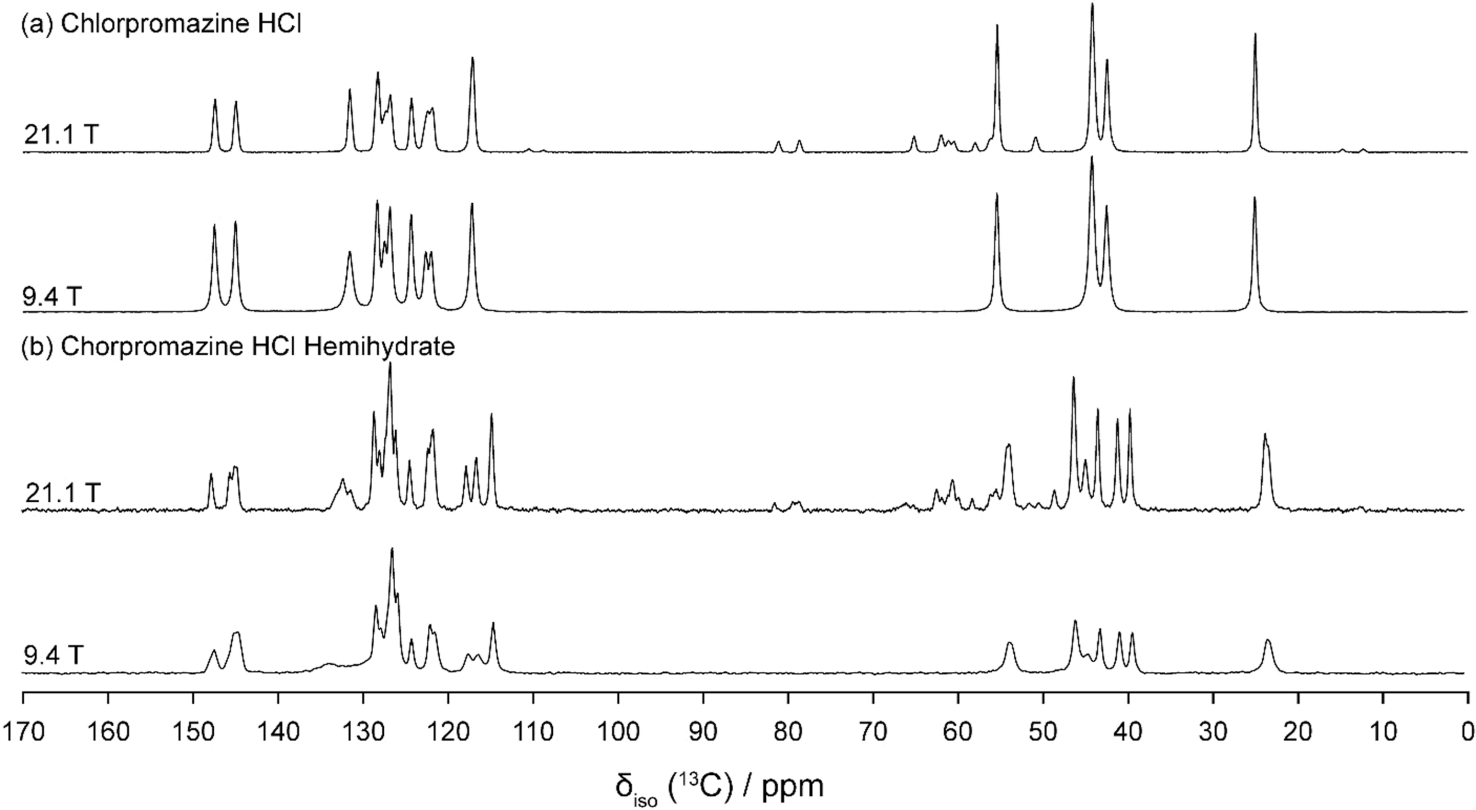
^13^C CP/MAS spectra of (a) chlorpromazine hydrochloride and (b) CPZ–HCl·1/2H_2_O acquired at 9.4 and 21.1 T.

Two-dimensional correlation NMR spectroscopic methods are helpful for assigning resonances leading to detailed structure characterization. To assign all ^13^C, ^1^H, and ^14^N signals for CPZ–HCl, we combined ^1^H DQSQ, ^1^H–^13^C HETCOR, and ^14^N–^13^C HMQC experiments.

The ^1^H DQ/SQ spectrum (Figure 4a) reveals several hydrogen connectivities. The horizontal cross-peaks and plane-wave DFT data allow a partial mapping of the structural elements involving hydrogen atoms. We can then use the ^1^H DQ/SQ data to aid in the interpretation of ^1^H–^13^C HETCOR data to identify connected carbon resonances (Figure 4b).

**Figure 4:**
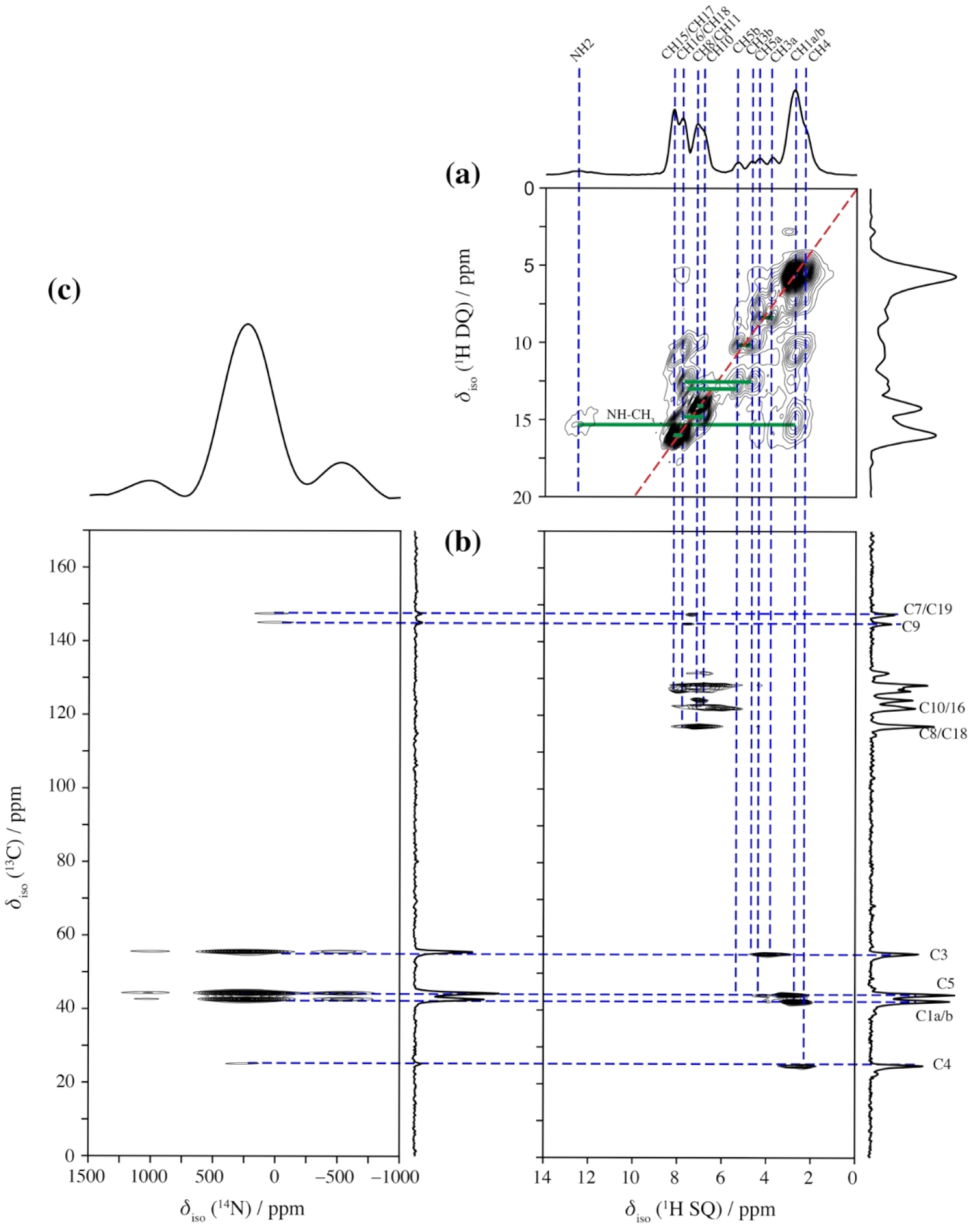
(a) ^1^H DQ/SQ, (b) ^13^C­[^1^H] HETCOR, and (c) ^13^C­[^14^N] TRAPDOR-HMQC experiments acquired for chlorpromazine hydrochloride. Assignments are labeled by the numbering scheme in Scheme 1. The notation a/b denotes either the two different protons in the respective CH_2_ groups, or the two different carbons in the two CH_3_ groups. The green lines in indicate short-range ^1^H–^1^H correlations in the DQ dimension. Blue dashed lines show correlations between the ^1^H, ^13^C and ^14^N sites. A projection of the ^1^H SQ dimension is shown above panel (a).

The ^1^H DQ/SQ spectrum reveals an area corresponding to the side-chain region of the chlorpromazine molecule, showing a pattern of correlations that suggests the presence of two side-chain configurations. Figure 5 presents an overlay of ^1^H DQ/SQ correlations from the DFT-optimized crystal structures of configurations I (blue) and II (red). While individual resonances cannot always be unambiguously assigned due to line broadening, systematic elongation of cross-peaks, particularly in regions where DFT predicts conformational differences, suggests the presence of two configurations (purple circles, Figure 5). On the right, intermolecular correlations between methyl protons and H16, differ by 1.6 kHz in the ^1^H coupling between configurations I and II. In the center, intramolecular side-chain correlations caused by slight shifts in the dihedral angles translate into small changes in the dipolar couplings and appear as elongation along the vertical or diagonal axes. By contrast, the geometry optimized single-conformation crystal structure of Dorignac-Calas yields a distinct pattern of DQ/SQ correlations that fails to reproduce these stretched features and was therefore excluded as a viable solution. Overall, the data confirms that at least two unique configurations underlie the structural disorder observed by SC-XRD.

**Figure 5:**
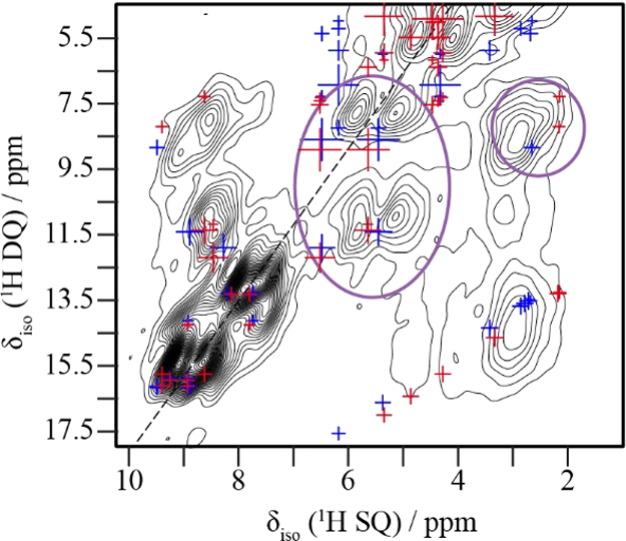
Enlarged area of the ^1^H DQ/SQ spectrum showing spectral features of interest. The blue and red crosses correspond to ^1^H correlations calculated by DFT for configurations I and II, respectively.

CPZ–HCl contains two nitrogen sites: one tertiary-substituted phenothiazine nitrogen and one protonated side chain nitrogen. This is verified using ^14^N WURST-QCPMG, where the spectrum presented in Figure 6a clearly shows two distinct quadrupolar powder patterns typical of spin-1 nuclei. The extracted quadrupolar NMR parameters are shown in Table 4, along with the DFT predictions. The neutral phenothiazine nitrogen exhibits a |*C*_Q_| of 5.04 MHz, while the protonated side chain amine has a |*C*_Q_| of 1.49 MHz, both near axial symmetry with η_Q_ = 0.07 and 0.09, respectively. These results agree with our DFT calculations and prior experimental trends^57^ predicting similar |*C*_Q_| and η_Q_ values, although, we cannot resolve two independent ^14^N signals corresponding to side chain configurations I and II. This suggests that the local bonding environment at the protonated side chain amine is nearly identical for both configurations, and the structural differences between the two configurations are manifested strictly at carbons C3, C4, and C5.

**Figure 6:**
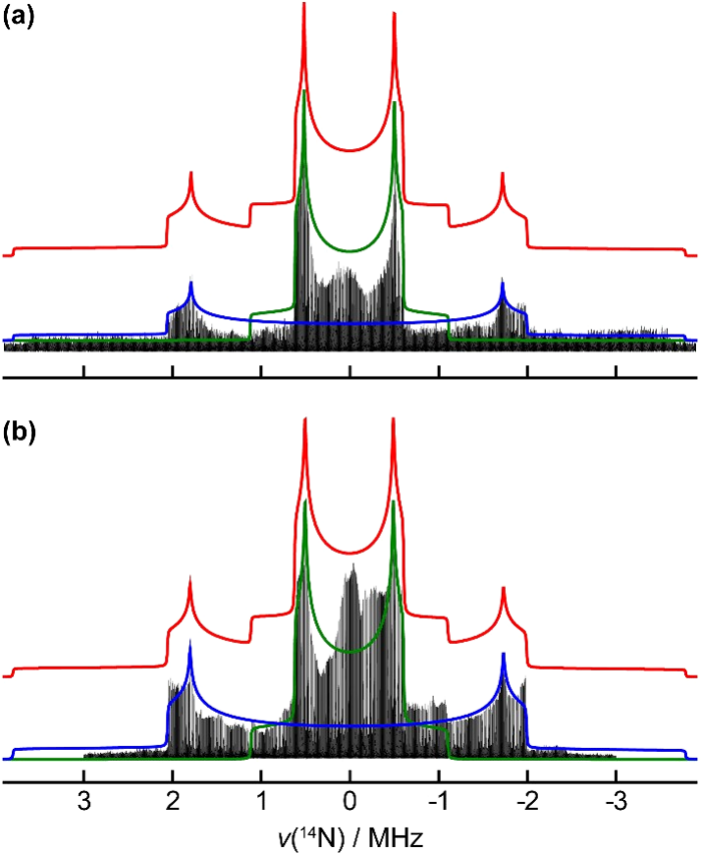
Static ^14^N WURST-QCPMG spectra of (a) CPZ–HCl and (b) CPZ–HCl·1/2H_2_O. The spectra were acquired by the VOCS method. Each spectrum piece was coadded to form the ultrawide line spectrum. Quadrupolar NMR parameters were extracted from the spectrum using QUEST,^58^ and the simulated line shapes for each site are overlaid in green and blue, and the combined sites in red.

**Table 4:** Calculated vs Experimental ^14^N NMR Parameters for CPZ–HCl and CPZ–HCl·1/2H_2_O

			CPZ–HCl						CPZ–HCl·1/2H_2_O					
			Expt.		*d* = 5; *s_6_* = 1.00		*d* = 3.25; *s_6_* = 1.00		Expt.		*d* = 5; *s_6_* = 1.00		*d* = 3.25; *s_6_* = 1.00	
			**I**	**II**	**I**	**II**	**I**	**II**	**A**	**B**	**A**	**B**	**A**	**B**
^14^N	cation	δ_iso_/ppm^a^	90		186.56	184.95	201.39	200.06	90		182.46	183.75	196.75	197.94
		*C*_Q_/MHz	1.49		–1.66	1.76	–1.66	1.74	1.48		1.85	1.58	1.88	1.67
		η_Q_	0.09		0.13	0.09	0.11	0.08	0.11		0.02	0.09	0.01	0.08
	*neut.*	δ_iso_/ppm^a^	100		122.80	120.40	129.01	127.64	100		116.38	117.67	123.80	124.74
		*C*_Q_/MHz	5.04		5.42	4.90	–5.18	4.72	5.05		4.91	4.95	4.77	4.83
		η_Q_	0.07		0.03	0.05	0.02	0.05	0.07		0.01	0.01	0.02	0.01

a Calculated values are given as magnetic shielding (σ_iso_) constants.

To correlate ^13^C sites to ^14^N, we also acquired a 2D ^13^C­{^14^N} TRAPDOR-HMQC experiment (Figure 4c). Two prominent correlations appear at 145 and 147 ppm, correlating with minor resonances in the ^13^C {^1^H) HETCOR. We attribute these resonances to phenothiazine carbons C19 and C7. We also observe more intense correlations for side chain carbons bound to the cationic ^14^N, including the methyl groups at 42 ppm, and C3 at 55 ppm. A signal at 44 ppm is correlated to C5, adjacent to the phenothiazine structure.

Overall, the correlation data presented in Figure 4 enable us to map out and confirm the ^13^C and some of the ^1^H chemical shift assignments. Table 5 shows the experimental ^13^C chemical shifts and DFT predicted isotropic shielding values. The corresponding plot in Figure 7a shows excellent correlation (*R* ^2^ = 0.9993) between the experimental shifts and GIPAW-predicted isotropic shieldings. In addition, the ^1^H DQ/SQ spectrum patterns have enabled us to confirm two distinct side-chain configurations, manifested as disorder by SC-XRD. Given that this disorder was not observed for the hydrate of CPZ–HCl, we endeavored to conduct a similar analysis.

**Table 5:** Calculated vs. Experimental ^13^C Chemical Shifts of Chlorpromazine Hydrochloride and Chlorpromazine Hydrochloride Hemihydrate

	Chlorpromazine Hydrochloride		Chlorpromazine Hydrochloride Hemihydrate (Molecule I)		*Chlorpromazine Hydrochloride Hemihydrate (Molecule II)*	
Atom number	*σ*_iso_ (^13^C) (calc.) [ppm]	*δ*_iso_ (^13^C) (expt.) [ppm]	*σ*_iso_ (^13^C) (calc.) [ppm]	*δ*_iso_ (^13^C) (expt.) [ppm]	*σ*_iso_ (^13^C) (calc.) [ppm]	*δ*_iso_ (^13^C) (expt.) [ppm]
1a/b	135.7	42.2	137.1	42.5	133.3	43.9
	137.8		141.1	38.6	139.7	40.1
3	124.2	55.1	124.9	52.9	125.0	52.9
4	152.6	24.8	157.0	22.7	157.3	22.7
5	132.4	44.0	131.1	45.3	132.7	45.3
7	28.7	147.1	27.1	146.9	29.9	143.9
8	57.5	116.9	59.8	116.9	58.3	116.9
9	38.2	131.3	38.1	131.4	36.1	131.4
10	52.7	121.7	53.5	120.8	53.6	120.8
11	47.6	127.2	47.7	125.8	48.9	125.2
12	45.5	128.0	45.7	127.7	46.3	127.1
14	44.0	128.0	45.4	130.5	45.5	127.7
15	47.7	127.2	47.3	125.8	46.7	126.3
16	51.8	124.0	50.6	121.4	53.2	120.8
17	48.3	126.5	49.0	123.5	48.3	125.2
18	58.7	116.9	61.1	115.7	61.2	113.9
19	29.3	144.7	29.7	144.2	28.8	144.7

**Figure 7:**
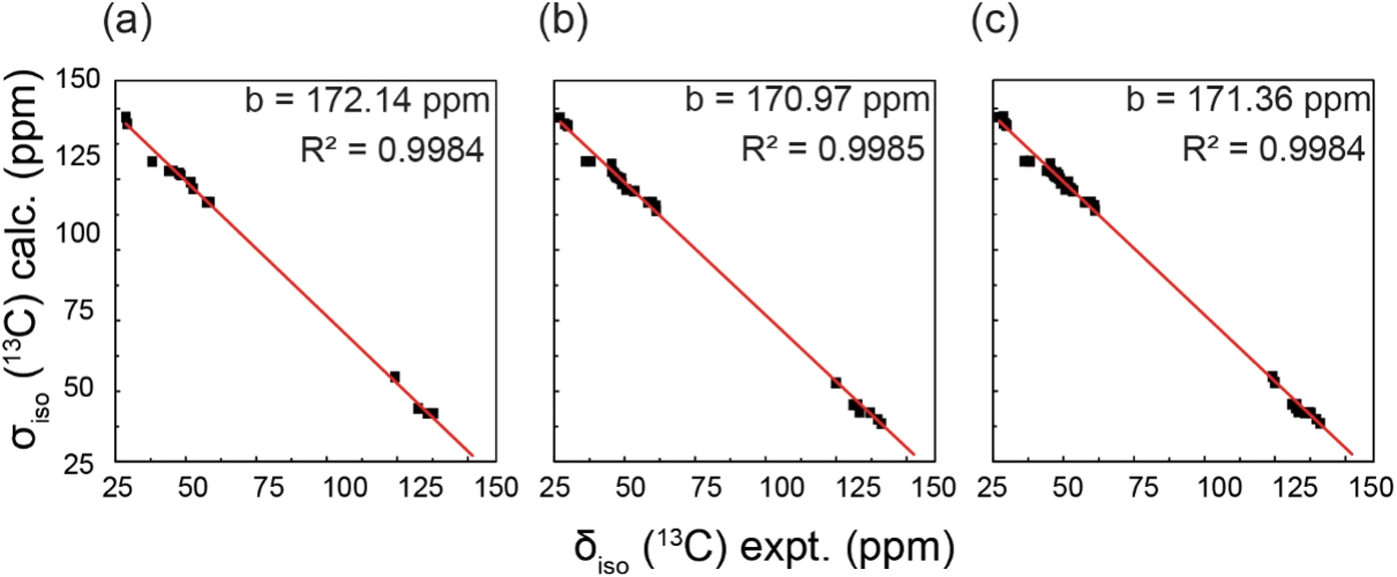
Correlation between calculated ^13^C isotropic shielding and experimental chemical shift shifts (a) and CPZ–HCl, (b) CPZ–HCl·1/2H_2_O, and (c) both compounds combined.

Disorder in aliphatic chains can sometimes appear as asymmetric doublets or shoulders to major ^13^C peaks, especially when cooling the sample to slow dynamic processes.^27,59^ Variable temperature CP/MAS experiments are well-known to aid in elucidating dynamics in crystalline species. The NMR data discussed so far indicate that the observed disorder arises from two distinct side-chain configurations (I and II), which coexist in a certain statistical distribution. Because we did not observe characteristic shoulders in the room temperature ^13^C spectra (Figure 3), cooling the sample down would be expected to resolve the peaks corresponding to each configuration of the side chain if dynamics are responsible for the disorder. Figure 8 shows the overlaid spectra for CPZ–HCl acquired from between 236 and 288 K in four steps. Interestingly, only the C5 signal resolved into a prominent peak and a shoulder at lower temperature, while the other resonances broaden and shift slightly to lower chemical shifts. Given that C5 is not crystallographically disordered, we attribute this splitting (∼60 Hz at 235 K) to residual dipolar coupling with the adjacent ^14^N site^60^ which has a relatively large quadrupolar coupling (5.04 MHz). The splitting collapses into one narrow peak at higher temperatures because of local dynamics leading to a partial averaging of quadrupolar effects, and a more rapid ^14^N spin–lattice relaxation time at elevated temperatures.^61,62^

**Figure 8:**
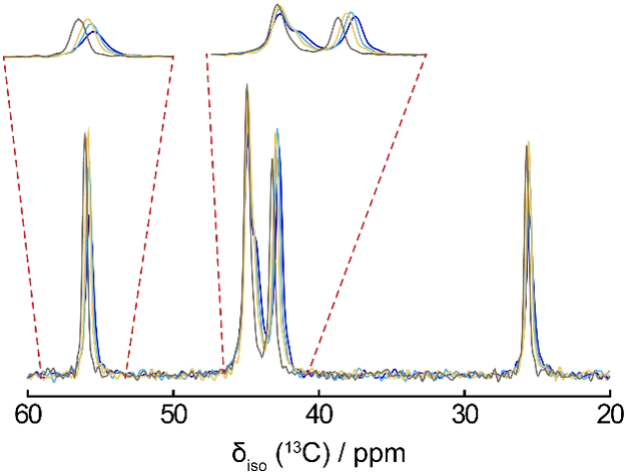
Variable temperature ^13^C CP/MAS NMR spectra acquired at 9.4 T for chlorpromazine hydrochloride (236–288 K). Expanded regions show the temperature dependent changes of peaks assigned to the dimethylaminopropyl side chain. Colors indicate different probe temperatures (blue at 235.6 K, light blue at 253.0 K, gold at 273.0 K and brown at 286.7 K).

A similar analysis of CPZ–HCl·1/2H_2_O (Figure 9) is complicated by the presence of two crystallographically unique molecules of chlorpromazine resulting in multiple correlations corresponding to the different molecules. Nonetheless, using GIPAW-calculated isotropic shielding values as a guide, we assigned all ^13^C and ^1^H resonances (Table 5 and Figure 7b), with excellent agreement between experimental and calculated data (*R* ^2^ = 0.9966). Notably, the ^1^H DQ/SQ spectrum (Figure 9a) did not show any evidence of side chain disorder, consistent with single-configuration crystallographic data.

**Figure 9:**
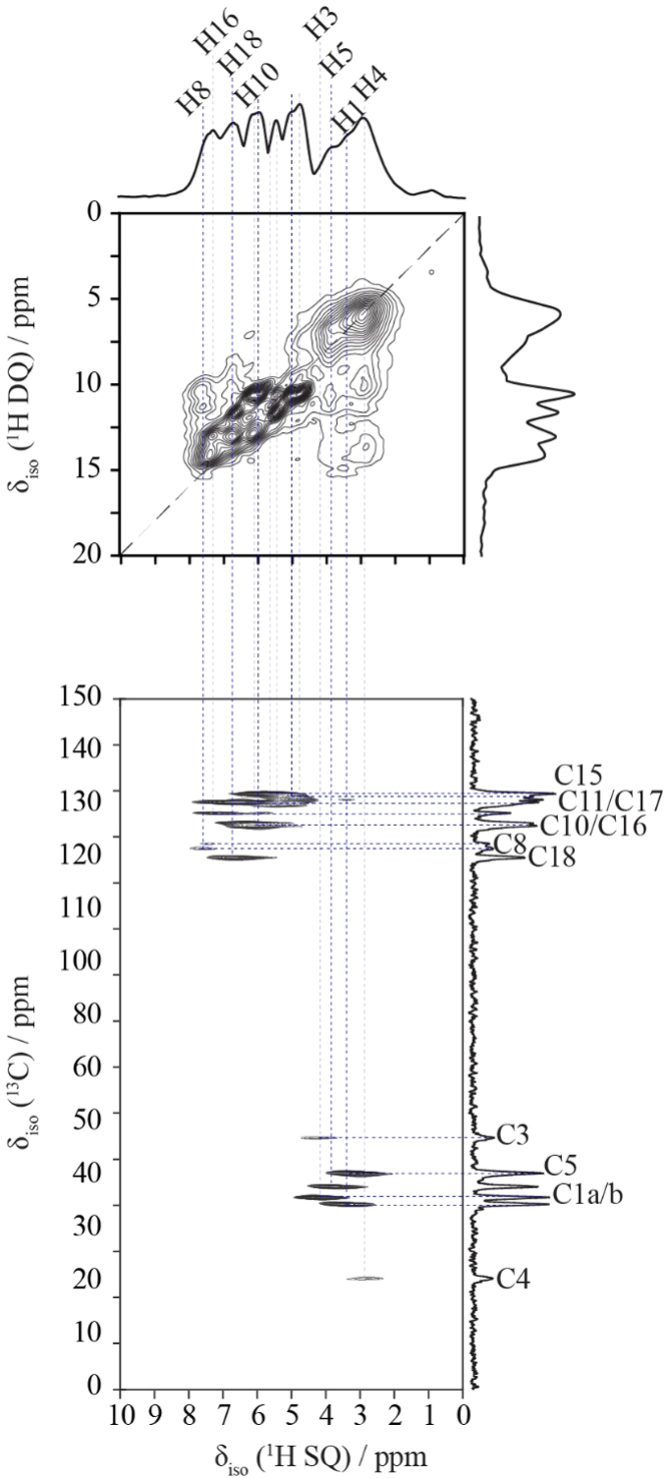
(a) ^1^H DQ/SQ and (b) ^13^C­[^1^H] HETCOR spectra acquired for chlorpromazine hydrochloride hemihydrate. Assignments are labeled by the numbering scheme in Scheme 1. Blue dashed lines show correlations between the ^1^H and ^13^C sites. For clarity, the NH_2_ signals and not all ^1^H chemical shift assignments are shown. See Table 5.

We attempted to acquire ^13^C­{^14^N} TRAPDOR-HMQC data for CPZ-HCl-1/2H_2_O, but were unsuccessful even after weeks of acquisition. The lack of correlation signals likely results from unfavorable longitudinal quadrupolar relaxation (*T*_1Q_) properties caused by the nearby water involved in the hydrogen bonding network at the side chain. Fast water dynamics can cause rapid fluctuations in the electric field gradient at the ^14^N nucleus, impeding effective coherence transfer in recoupling experiments. Hu and Schmidt–Rohr previously reported that fast ^14^N *T*_1Q_ relaxation resulted in significant difficulties in observing signals RE­(SP)­DOR experiments.^63^ Still, the static ^14^N WURST-QCPMG spectrum (Figure 6b) confirms two ^14^N sites: neutral and cationic. The values, |*C*_Q_| = 5.05 MHz and η_Q_ = 0.07 for the neutral ^14^N site, and |*C*_Q_| = 1.48 MHz and η_Q_ = 0.11 for the cationic site, agree very well with the anhydrous form of CPZ–HCl. Given the two crystallographically distinct chlorpromazine molecules exhibit nearly identical ^14^N quadrupolar parameters, according to our DFT predictions (Table 4), we could not experimentally resolve all four ^14^N sites.

For both the hydrochloride and the hemihydrate, we made ^13^C chemical shift assignments for all resonances using plane wave DFT as a guide. The correlation of all experimental shifts to calculated shieldings is shown in Figure 7c. The overall validity of this method is confirmed by the high *R* ^2^ value of 0.9968, indicating that most chemical shifts were accurately predicted using plane wave DFT.

### ^35^Cl NMR Spectroscopy

To probe the electronic environment immediately surrounding the chloride anions, we acquired both static and magic angle spinning ^35^Cl spectra at *B*_0_ = 21.1 T for CPZ–HCl and its hemihydrate (Figure 10). The resulting lineshapes appear as central transition powder patterns with clear shapes and excellent signal-to-noise. The experimental and DFT-predicted parameters are shown in Table 6. According to the crystal structures (Table 2), the distance between the chlorine anion and the cationic amine is 2.96 Å for CPZ–HCl. Additionally, the chloride anion also forms short contacts with the side chain methyl and methylene carbons with distances between ∼2.7–2.9 Å in configuration II, but these are absent in configuration I.

**Figure 10:**
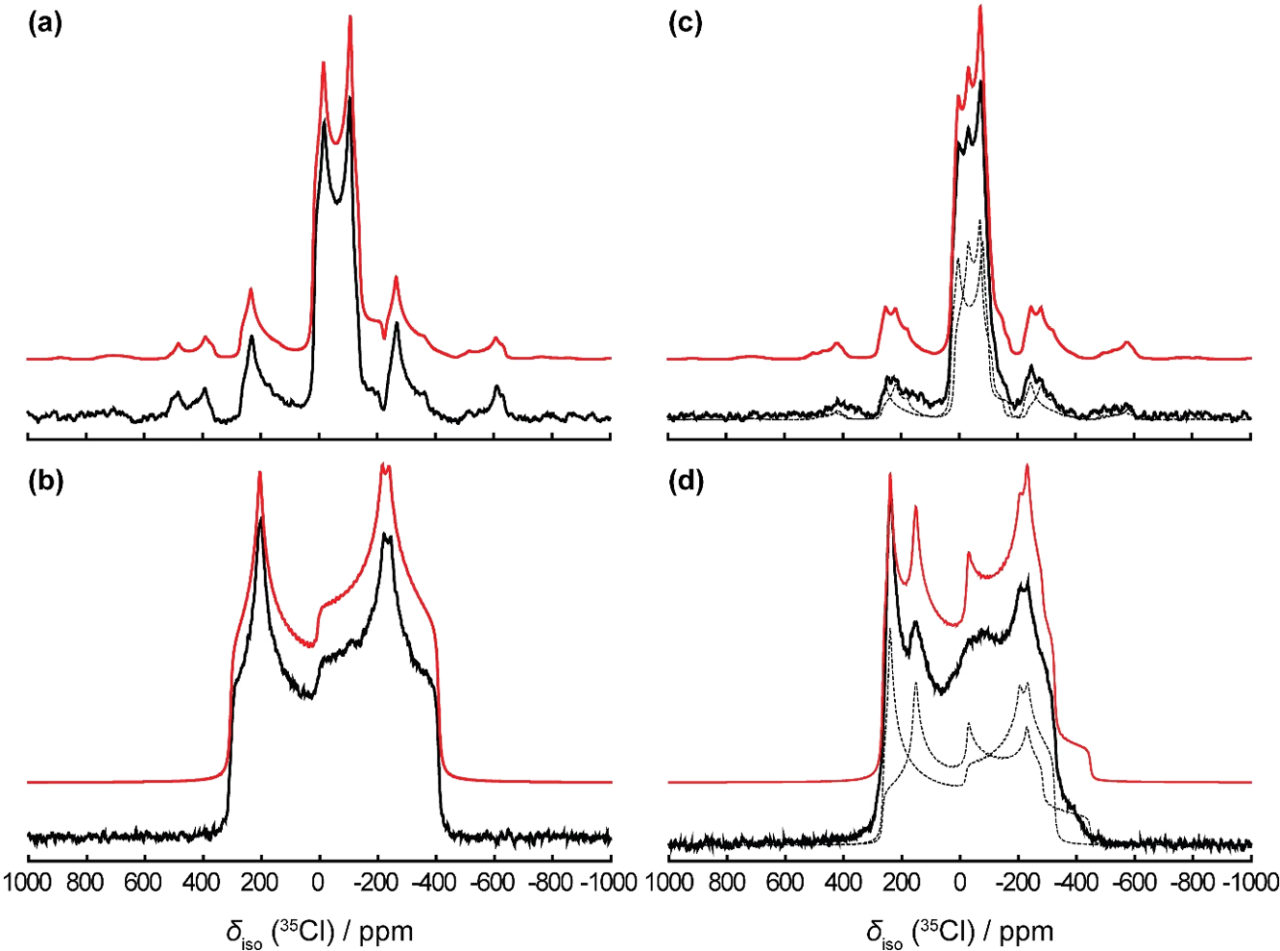
^35^Cl NMR spectra of chloride ions in chlorpromazine hydrochloride (a, b) and the hemihydrate (c, d). MAS (a, c) spectra are compared to static spectra (b, d). The simulated line shapes are overlaid in red.

**Table 6:** Experimental and Calculated ^35^Cl NMR Parameters for CPZ–HCl and CPZ–HCl·1/2H_2_O

			CPZ–HCl						CPZ–HCl·1/2H_2_O					
			Expt.		*d* = 5; *s_6_* = 1.00		*d* = 3.25; *s_6_* = 1.00		Expt.		*d* = 5; *s_6_* = 1.00		*d* = 3.25; *s_6_* = 1.00	
			**I**	**II**	**I**	**II**	**I**	**II**	**A**	**B**	**A**	**B**	**A**	**B**
^35^Cl	*anion*	δ_iso_/ppm^a^	41.18		42.94	46.84	15.86	18.56	23	49	88.72	1.6.19	72.72	78.75
		*|C*_Q_|/MHz	5.67		6.12	6.96	4.58	5.23	4.58	5.25	6.24	5.81	5.07	4.3
		η_Q_	0.25		0.10	0.13	0.12	0.12	0.50	0.20	0.71	0.76	0.73	0.41
	*cov.*	δ_iso_/ppm^a^	350.00		309.78	307.36	277.00	275.2	Not observed		329.44	355.73	313.87	319.04
		*|C*_Q_|/MHz	67.80		66.48	66.76	65.53	65.69			67.05	68.46	66.15	67.49
		η_Q_	0.09		0.11	0.11	0.12	0.12			0.08	0.12	0.08	0.12

a Calculated values are converted from isotropic magnetic shielding (σ_iso_) constants to chemical shift (δ_iso_) values using the formula δ_iso_ = σ_ref_ – σ_iso_, where σ_ref_ = 962 ppm.^27^

For CPZ–HCl, the DFT predictions suggest that the chloride anion experiences slightly different EFG parameters for configurations I and II. Configuration I, having no short contacts with the side chain, has a slightly smaller quadrupolar coupling constant. However, we only resolve a single site in the MAS spectrum (Figure 10a), with *C*_Q_ = 5.67 MHz and η_Q_ = 0.25, consistent with earlier measurements.^64–66^ The static powder pattern (Figure 10b), shows a split lower-frequency horn, and a very sharp higher-frequency horn, but only a single discontinuity in the middle. Our fit model therefore assumes a single ^35^Cl site affected by anisotropy in the chemical shift tensor. Our best fit yields quadrupolar parameters of *C*_Q_ = 5.67 MHz and η_Q_ = 0.25, and chemical shift tensor parameter δ_iso_ = 41.18 ppm, δ_aniso_ = −42.65 ppm and η_aniso_ = 1.0. Euler angles affected the powder pattern’s shape significantly with β, the angle between *V*_33_ and δ_33_, having a dominant effect on the magnitude of the splitting in the lower-frequency horn. The best fit used Euler angle values of α = −46.7°, β = 11.23°, and γ = −62.03°.

The single-site behavior of the ^35^Cl anion indicates that its local environment is nearly the same relative to both side chain configurations. The side chain is anchored in place by the N–H···Cl^–^ hydrogen bond, which has an outsized impact on the ^35^Cl EFG tensor parameters. Additionally, the data also suggests that no C–H···Cl^–^ interactions occur at C3 and C4, allowing these sites to adopt structural flexibility. The ionic chlorine is unperturbed by differences in the alkyl hydrogen positions between the two configurations.

In contrast, the hemihydrate has two crystallographically unique chloride anions. Based on the SC-XRD structure, one is engaged in hydrogen with the N–H of one chlorpromazine molecule and the water molecule. The N–Cl and O–Cl distances are 3.1 and 3.2 Å, respectively, and the N–Cl–O angle is 85°. The chloride anion also interacts with a second water molecule, at a distance of 3.29 Å and an N–Cl–O angle of 151°. The other chloride anion only engages in a single N–H hydrogen bond, with an N–Cl distance of 3.0 Å. The MAS spectrum (Figure 10c) shows two sites, A (*C*_Q_ = −4.58 MHz, η_Q_ *=* 0.5) and B (*C*_Q_ = 5.25 MHz, η_Q_= 0.20).

The static spectrum (Figure 10d) shows two sites, with a broadened feature in the center of the powder pattern. Site A has a larger chemical shift anisotropy (δ_iso_ = 49 ppm, δ_aniso_ = −75 ppm, η_aniso_ = 0.25, α = 90, β = 8, and γ = 90°) than site B (δ_iso_ = 23 ppm, δ_aniso_ = −185 ppm, η_aniso_ = 0.52, α = 174, β = 31, and γ = 129°). The larger anisotropy at site A likely reflects a more complex bonding environment resulting from an asymmetric coordination to the two chlorpromazine molecules and water, while site B is characterized by a slight broadening in the higher frequency peak, which is likely due to the influence of the dynamics of the nearby water molecule. This has a more significant effect on the lower frequency horn, which is manifested as the broad hump overlapping the discontinuity in the powder pattern of site A, making it challenging to fit with certainty. Site B more closely resembles the powder pattern of CPZ–HCl, indicating similar N–H···Cl^–^ hydrogen bonding.

The DFT geometry optimizations with two-body dispersion reparameterization of *d* = 5 best reproduce the X-ray structure (Table 3), but they overestimate the ^35^Cl quadrupolar coupling and asymmetry parameters (Table 6). The reverse is true when *d* = 3.25 is used, although the agreement to the experimental NMR parameters is closer. This discrepancy likely arises because while reparameterizations may improve geometries around carbon and hydrogen atoms, they may not optimally describe the wave function for heavier atoms such as chlorides, an issue that becomes more obvious during the calculations of the NMR properties.

In addition to the ionic chlorines, chlorpromazine features a covalently bound chlorine at C9 of the phenothiazine structure. Covalent chlorines typically exhibit very large quadrupolar couplings resulting in very wide central transition line widths, making them are notoriously difficult to study by conventional ^35^Cl solid-state NMR techniques. Indeed, relatively few ^35^Cl studies have been carried out to observe covalent chlorines,^64,65,67–70^ with nuclear quadrupole resonance (NQR) spectroscopy sometimes taking its place.^71^ However, there is a great interest in studying covalent chlorines due to their importance in biological and chemical reactions and, recently, halogen bonding.^72^ To date, the WURST-QCMPG pulse sequence^40,41^ has provided one of the most efficient ways to collect ultrawide line ^35^Cl spectra, allowing for spectra acquisition over hours to days. However, our attempts for both chlorpromazine hydrochloride and the hydrate but were unsuccessful because a *T*_2_ of ∼0.11 ms resulted in the echo decaying entirely after the first QCPMG pulse. Instead, a quadrupolar echo experiment was used at variable offsets for CPZ–HCl selectively capturing the shapes of the high- and low-frequency edges of the powder pattern (Figure 11) Fitting the skyline projection and summation spectrum of the powder pattern yields *C*_Q_ = −67.8 MHz and η_Q_ = 0.09, consistent with literature examples of covalent chlorines bonded to aromatic ring structures.^69^ Similar experiments for the hemihydrate were impractical due to the excessively long experimental times (>1 week) and were not carried out.

**Figure 11:**
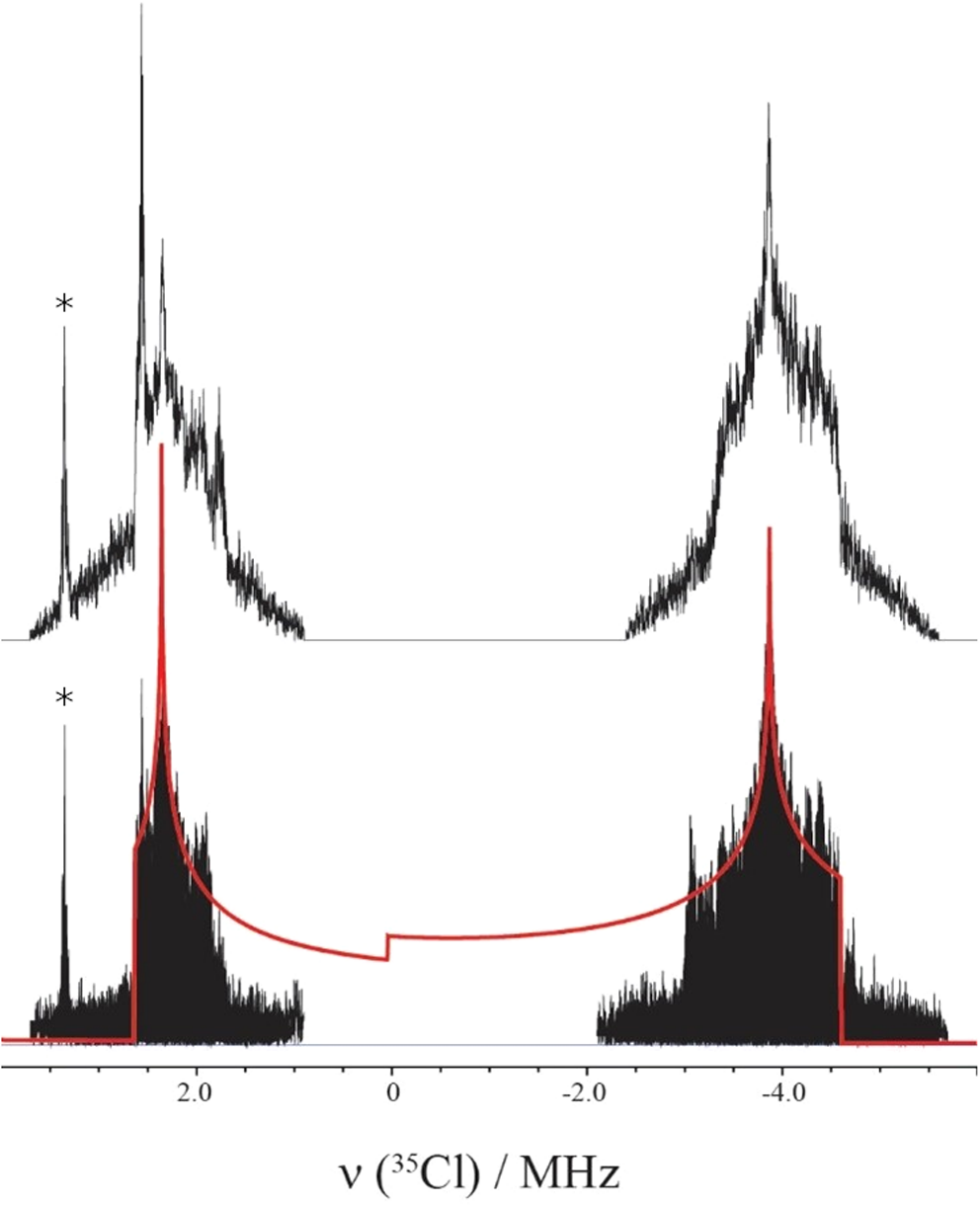
Static ^35^Cl quadrupolar echo spectrum of covalent chlorine in chlorpromazine hydrochloride, acquired by the VOCS method. The summation (top) and the skyline projection (bottom) of multiple pieces collected over a range of frequencies. The simulated line shape is overlaid in red. The asterisks mark known radio station frequencies.

### Molecular Dynamics Simulations

**Figure 12:**
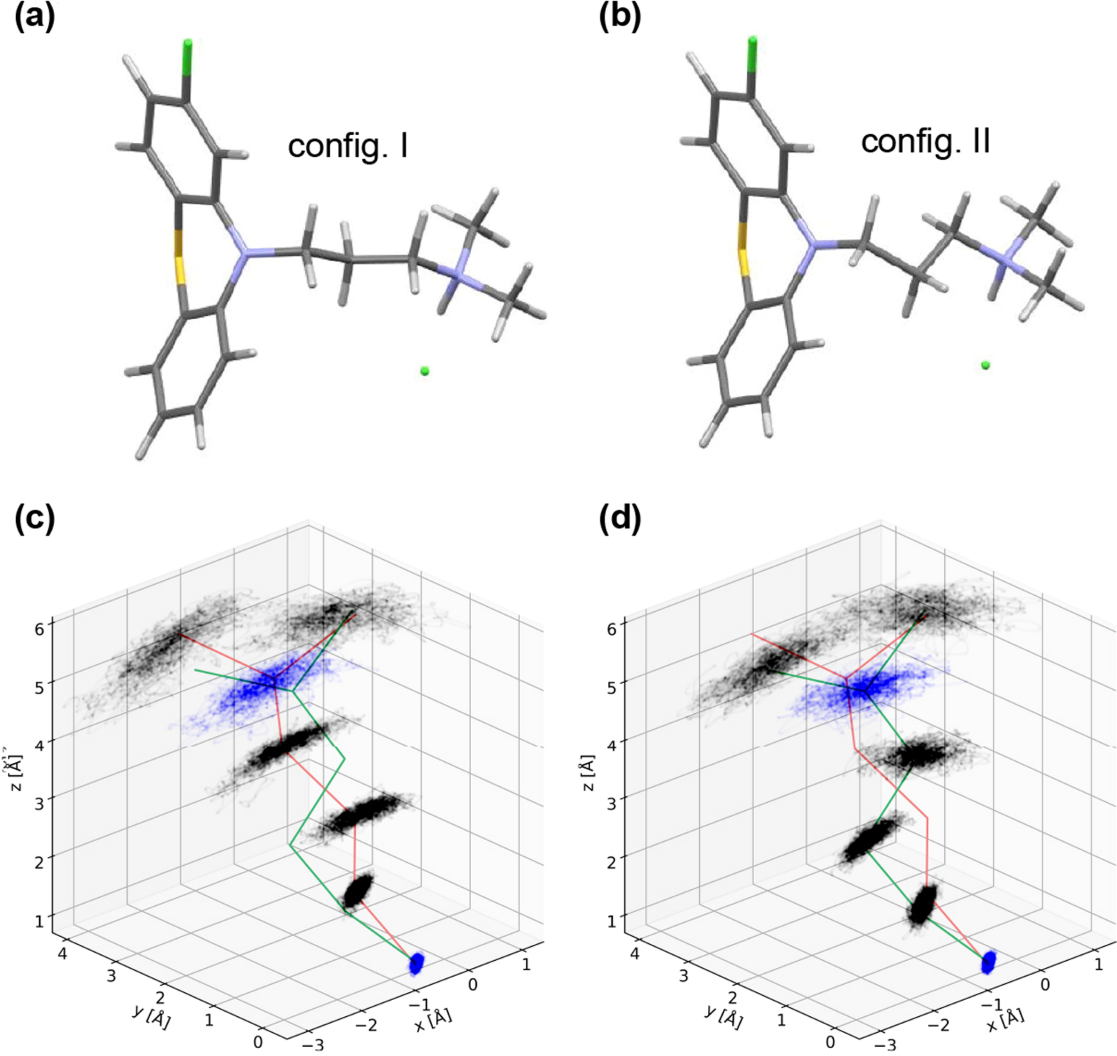
MD simulations of the side-chain region of CPZ–HCl starting from configurations I ((a) and (c)), and II ((b) and (d)). The bottom plots show the positions adopted by the side-chain atoms as “clouds.” The initial configurations are shown in orange (I) and green (II) to demonstrate that the configurations do not transition from one to the other.

The variable temperature ^13^C spectra (Figure 8) indicate that the side chain undergoes a degree of motion with changing temperature. However, a careful comparison of the room temperature ^1^H SQ/DQ spectrum (Figure 3) with DFT calculated ^1^H chemical shifts reveals multiple correlations corresponding to the protons at positions C3 and C4, suggesting that the side-chain predominantly adopts configurations I and II in the form of static disorder rather than dynamically. By contrast, the ^35^Cl data can be modeled using a single set of EFG tensor parameters, suggesting that either the chloride atom experiences effectively identical local environments in both configurations, likely acting as a hydrogen-bond anchor, or that rapid dynamics might obscure any distinction between each configuration.

To determine which hypothesis is correct, we performed molecular molecular dynamics simulations using configurations I and II as a starting points (Figure 12). Previous work showed a ∼16.3 kJ/mol activation energy for side chain motion in neutral chlorpromazine^22^, while Hensel et al. reported an activation energy of 27.2 kJ/mol attributed to the γ-relaxation process in CPZ–HCl (dimethylamine group rotation), but this does not address the overall configuration of the side chain.^73^ Here, we find that although local motions occur in the side chain, it is not likely to hop between configurations I and II. A DFT transition state search (Figure 13) predicts a barrier of 14.41 kJ/mol between configurations I and II, with configuration II being slightly more stable, likely due to a lower energy torsional geometry. Qualitatively, the ^1^H SQ/DQ data (Figure 4) indicates as higher signal intensity for one side-chain configuration, which we attribute to the increased probability of the structure adopting configuration II. In comparison to neutral chlorpromazine, the much larger barrier reflects the additional N–H···Cl^–^ hydrogen bond anchoring the side chain in place, creating static disorder.

**Figure 13:**
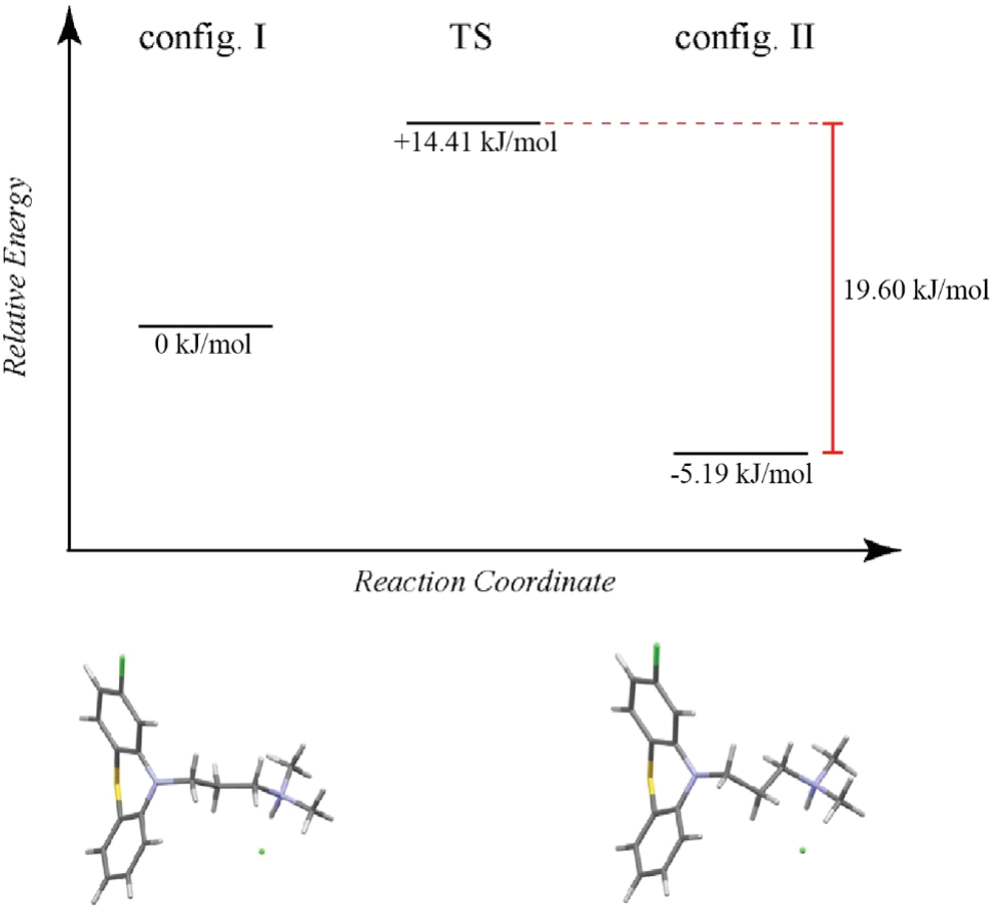
Relative energy diagram from a TS search showing the equilibrium between configurations I and II of the dimethylaminopropyl side chain in chlorpromazine hydrochloride. The relative energy of configuration I is set to 0 kJ/mol. Refer to Figure 2b for a depiction of the unit cell used in the TS calculation.

## Conclusions

We have carried out a detailed NMR crystallographic study of chlorpromazine HCl and its hemihydrate. For the hydrochloride salt, a combination of solid-state NMR, DFT, and MD simulations provides valuable insights into how the 3-(dimethylamino)­propyl side chain adopts two distinct configurations. Transition state search and MD simulations reveal a large (∼14 kJ/mol) barrier separating these configurations, confirming that the side chain configuration does not easily interconvert at room temperature. In contrast, the hemihydrate crystallizes with a single stable conformation of the side chain, presumably due to water-mediated hydrogen bonding restricting the conformational space.

Our ^14^N and ^35^Cl NMR data highlight how hydrogen bonding to the chloride anion and dynamic water molecules affect local structure and relaxation properties. While covalent chlorines remain challenging to study by ^35^Cl NMR due to short relaxation times and large quadrupolar couplings, partial spectra can be obtained through multistep echo experiments.

Overall, this work demonstrates how NMR crystallography can elucidate subtle structural disorder characteristics in APIs. Understanding such disorder is critical for designing robust pharmaceutical forms and predicting their physicochemical properties. As crystal structure prediction methods evolve, a deeper knowledge of static and dynamic disorder, including relevant hydrogen bonding and molecular motions, can further refine computational approaches to drug development.

## §

Eli Lilly, Kinsale, Ireland
